# Editorial: Molecular Diagnostics in the Detection of Neurodegenerative Disorders

**DOI:** 10.3389/fmolb.2017.00010

**Published:** 2017-03-02

**Authors:** Megha Agrawal, William C. Cho

**Affiliations:** ^1^Department of Bioengineering, University of Illinois at ChicagoChicago, IL, USA; ^2^Department of Clinical Oncology, Queen Elizabeth HospitalKowloon, Hong Kong

**Keywords:** Alzheimer's disease, biomarkers, neurons, Parkinson's disease, stem cells

Neurodegenerative disorders encompass a range of medical conditions which primarily affect the neurons in the human brains. Such conditions result in the disorders of the central nervous system which eventually lead to the progressive loss of neural tissues including death of neurons (Agrawal and Biswas, 2015). An impressive volume of research has been conducted over the last few decades to advance our understanding of this fatal disease. The challenge to treat neurodegenerative disorders lies in the inability of the neurons to regenerate on their own once they start functioning abnormally after the neural deterioration or once a severe damage occurs to a neural tissue. However, stem cell therapy has been proven to be potentially useful in neuroregeneration or even neuronal cell replacement (Chung et al., 2002; Rachakonda et al., 2004). One of the most important missions of diagnosis and prognosis of neurodegeneration is the ability of early detection of the onset of neurodegeneration. An early diagnosis of the disease is critical as it provides a chance for an early treatment that may be helpful to prevent further progression of the deadly neurodegeneration and its aftermaths that takes millions of lives every year globally (Miller and O'Callaghan, 2015).

With an aim to provide a discussion platform to neurologists, neuroscientists and pathologists for sharing the latest findings and knowledge on neurodegeneration and the molecular diagnostics to detect and combat neurodegeneration, we have launched this special research topic on molecular diagnostics in the detection of neurodegenerative disorders. We anticipate that molecular diagnostics will play an imperative role in near future for providing an effective diagnostic solution to the complex problem of neurodegenerative diseases. Based on the available research data, we firmly believe that molecular diagnostics can be effective to detect and diagnose various neurological diseases such as Amyotrophic lateral sclerosis, Huntington's, Alzheimer's, and Parkinson's disease, at an early stage (Gasser et al., 2001a,b, 2003; Agrawal and Biswas, 2015). Molecular diagnostics in neurodegenerative disorder is an emergent area of study, research and development. It represents a multidisciplinary research field that offers plenty of opportunities for collaboration between neurologists, psychologists, biologist and biomaterials scientists and other trained personnel with the necessary experience in managing the diseases. We expect that further developments in various molecular diagnostics will pave the way for the early detection of neurodegeneration and effective treatment.

This e-book showcases important and significant reports that cover a wide range of areas in neurodegeneration research and treatment. These include diagnosis and prognosis; role of neuroactive drugs in regulating central nervous system; advances in novel biomarkers; brain injury induced neurobehavioral outcomes and also connectivity between spinal muscular atrophy and loss of α-motors neurons among other reports. One of the reports investigates the origin and potential function of corpora amylacea (CA) which are found in large numbers in the central nervous system of the patients with neurodegenerative diseases. Immunohistochemistry analyses were employed to reveal fungal proteins present in CA from patients diagnosed with Alzheimer's disease (Pisa et al.). An insight into the prospective roles of haptoglobin (Hp) (an endogenous hemoglobin-binding protein) in traumatic brain injury and other acute brain injuries is discussed in another report which portrays to be helpful in understanding the inconsistency in outcomes of clinical studies regarding the importance of Hp phenotypes in such brain injuries (Glushakov et al.). This study is a step forward to develop and progress with new therapeutics in the prevention of cerebral hemorrhage which is a common feature of traumatic brain injury and its associated chronic disabilities (Glushakov et al.). Furthermore, important and significant biomarkers for neurodegeneration have been investigated and studied for their sensitivity and specificity in this e-book that sheds new lights in treatment of irreversible cognitive deficit and dementia in elderly population. Biomarkers represent important molecular diagnostic tools and thus the development of novel biomarkers could bring significant breakthroughs in an early diagnosis of neurodegeneration (Sfera et al.).

In a particular review, the application of stem cells and induced pluripotent stem cells in combating neurodegeneration is discussed that addresses the issues of diagnosis, modeling, and therapeutic transplantation strategies (Singh et al.). In another review, adropin is discussed as a biomarker for the diagnosis of central nervous system disorders and is considered as a potential therapeutic candidate in central nervous system injuries (Shahjouei et al.). It is believed that hyper excitability in neuronal network possibly contributes to the cognitive deficits in Alzheimer's disease and this is addressed in a research report that sheds light on the mechanism of aberrant neuronal networks in Alzheimer's disease (Wang et al.). To this end, researchers conducted investigations on the excitability in cultured pyramidal neurons from APP/PS1 mice using patch clamp recording techniques. This provides an important insight into the pathogenesis of Alzheimer's disease that could be helpful in developing new therapeutic avenues in the future (Wang et al.). This e-book covers another area of neurodegeneration: spinal muscular atrophy, which is an early-onset, autosomal recessive neurodegenerative disease that is characterized by the loss of spinal α-motor neurons leading to infant deaths (Butchbach). The author highlights why a better understanding of the underlying mechanism causing spinal muscular atrophy and the accurate measurements thereof are crucial to control such neurodegenerative disease in infants (Butchbach). The molecular mechanisms of C9orf72 gene causing amyotrophic lateral sclerosis, frontotemporal dementias and atypical parkinsonian syndromes are also discussed (Munteanu and Lynch).

In concluding our thoughts and deliberations, we do hope that this discussion forum in the form of an edited e-book will advance our further understanding to have an enhanced insight of neurogenesis and eventual cure for neurodegenerative diseases. Future research directions might involve multifunctional biomolecular diagnostic markers and technology platforms that would significantly enhance and augment the accuracy, specificity and sensitivity that would drive an early diagnosis and prognosis of various neurodegenerative disorders.

## Author contributions

Both authors have made substantial intellectual contribution to the work, and approved it for publication.

### Conflict of interest statement

The authors declare that the research was conducted in the absence of any commercial or financial relationships that could be construed as a potential conflict of interest.
